# Quantitative Proteomics of Plasma-Derived Extracellular Vesicles Reveals a Plasminogen-Centered Protein Network in Transthyretin Cardiac Amyloidosis

**DOI:** 10.1016/j.jacadv.2026.103302

**Published:** 2026-10-08

**Authors:** Amira Zaroui, Damien Habert, Benoit Vallée, Mounira Kharoubi, Soraya Fellahi, Benoit Vingert, Michel Seve, Ilaria Cascone, Vincent Audard, Emmanuel Itti, Hajer Abroud, Arnault Galat, Thibaud Damy, Sandrine Bourgoin-Voillard, Valérie Molinier-Frenkel

**Affiliations:** a AP-HP (Assistance Publique-Hôpitaux de Paris), Hôpitaux Universitaires Henri Mondor, Département de Cardiologie, Creteil, France; b French Referral Center for Cardiac Amyloidosis, Cardiogen Network, GRC Amyloid Research, Creteil, France; c AP-HP, GRC Amyloid Research Institute, Henri Mondor University Medical Center, Créteil, France; d Ecole Doctorale Santé Publique, Université de Paris-Est Creteil, Créteil, France; e Clinical Epidemiology and Ageing (CEpiA) Geriatrics, Primary Care and Public Health, Henri Mondor University Medical Center, Créteil, France; f Univ. Paris Est Creteil, INSERM, Institut Mondor de Recherche Biomédicale (IMRB), Creteil, France; g AP-HP, Hôpitaux Universitaires Henri Mondor, Département de Biochimie-Toxicologie-Pharmacologie, Creteil, France; h Etablissement Français du Sang, Créteil, France; i Univ. Grenoble-Alpes, TIMC, CNRS UMR 5525, Grenoble INP, EPSP, Grenoble, France; j CHU Grenoble Alpes, Grenoble, France; k AP-HP, Hôpitaux universitaires Henri Mondor, Service de Néphrologie-Transplantation, Creteil, France; l AP-HP, Hôpitaux Universitaires Henri Mondor, Service de Biophysique et Médecine Nucléaire, Creteil, France; m AP-HP, Hôpitaux Universitaires Henri Mondor, Département d’Hématologie-Immunologie, Creteil, France

**Keywords:** amyloidosis, cardiomyopathy, extracellular vesicles, heart failure, proteomics, transthyretin

## Abstract

**Background:**

The prognosis of patients with transthyretin cardiac amyloidosis (ATTR) remains poor; this is primarily due to late diagnosis, when irreversible damage has already occurred. Circulating levels of transthyretin or other plasma markers have little value for diagnosis. Although extracellular vesicles (EVs) (key mediators of intercellular communication) may reflect disease-specific molecular changes, their protein cargo has not yet been explored in the context of ATTR cardiomyopathy (ATTR-CM).

**Objectives:**

We evaluated whether cardiac amyloidosis alters the proteome of circulating EVs and assessed whether these changes define signature of ATTR.

**Methods:**

Plasma EVs were prospectively isolated by ultracentrifugation from 41 patients with confirmed ATTR-CM (ATTR+ group; wild-type ATTR and variant ATTR) and 24 frequency-matched patients with nonamyloid cardiomyopathy (ATTR− group), all referred to the Referral Center for Cardiac Amyloidosis (Henri Mondor Hospital, Créteil, France). EVs were characterized by flow cytometry and analyzed by quantitative liquid chromatography mass spectrometry/mass spectrometry proteomics.

**Results:**

ATTR+ patients had a distinct signature enriched in proteins associated with vascular homeostasis, coagulation, and inflammation. At least 18 of these proteins formed an interconnected network centered on plasmin/plasminogen. Notably, EV levels of transthyretin and plasminogen were elevated, whereas the level of alpha2-antiplasmin (plasmin's primary inhibitor) was low. This imbalance is particularly relevant because plasmin is known to promote amyloidogenesis via transthyretin cleavage.

**Conclusions:**

These exploratory findings highlight a plasmin/plasminogen-centered protein network linking fibrinolysis, complement activation, and vascular inflammation to ATTR pathophysiology, and suggest that plasma EV proteomics warrant further evaluation as a minimally invasive approach for disease monitoring and biomarker discovery in ATTR-CM.

Amyloidosis encompasses a heterogeneous group of infiltrative diseases characterized by the extracellular deposition of insoluble fibrils. These fibrils are formed by the aggregation of misfolded autologous proteins, the nature of which defines the type of amyloidosis.^1^ One of the most prevalent forms of cardiac amyloidosis results from the deposition of transthyretin (TTR) either its wild-type or variant form. TTR is a homotetrameric plasma protein, but gene mutations and ageing can limit its clearance and/or facilitate dissociation of the tetramer, partial unfolding, and aggregation.^2^^,^^3^

TTR amyloidosis (ATTR) cardiomyopathy (CM) (ATTR-CM) is a restrictive CM with a poor prognosis, with life expectancy typically ranging from 2 to 6 years after diagnosis. Although ATTR-CM was once considered a rare disease, recent advances in diagnostic techniques have revealed that the prevalence has been significantly underestimated.^4^^,^^5^ The World Heart Federation acknowledges that improving survival in patients with ATTR-CM depends on early diagnosis, appropriate management, a deeper understanding of its pathophysiology, and the development of targeted therapies.

Mass spectrometry–based proteomic analyses of cardiac tissue samples has demonstrated its effectiveness in diagnosing ATTR-CM.^6^^,^^7^ The identification of ATTR-specific protein markers in minimally invasive biological samples of body fluids has remained largely elusive. Although a recent publication suggested promising findings,^8^ most studies failed to evidence ATTR-specific protein markers in minimally invasive samples of body fluids, such as blood.^9^ In particular, the plasma TTR concentration is not considered to be a relevant diagnostic biomarker per se.^10^

Extracellular vesicles (EVs) are fundamental mediators of intercellular communication; they influence cellular and molecular functions by transporting a variety of biomolecules, including proteins, lipids, and RNAs, released by specific cell types. Notably, EVs modulate complex biological processes (such as immune responses, cell proliferation, cell differentiation, and extracellular matrix (ECM) remodeling), depending on their functional interactions.^11^, ^12^, ^13^ Thus, a better understanding of the EVs’ functions might lead to many medical applications, such as the identification of new protein biomarkers for cardiovascular diseases.^14^, ^15^, ^16^, ^17^ Exploring EVs is particularly relevant in ATTR, where circulating biomarker levels do not fully capture the extent of tissue damage, disease activity, or organ-specific involvement. By reflecting the molecular state of their cells of origin, EVs may offer a more sensitive and biologically informative window into the underlying disease mechanisms. Even though TTR is present in circulating EVs and a prior small pilot study reported that EV proteomic composition may reflect the ATTR disease process or suggested a diagnostic value,^18^ this topic has not been explored previously in ATTR-CM encompassing both wild-type or variant ATTR (ATTRwt and ATTRv, respectively), or compared findings against nonamyloid CM.

We hypothesized that cardiac amyloidosis could alter the proteome of circulating EVs. Specifically, we suspected that EVs released by cells stressed by modifications of their microenvironment present specific molecular signatures reflecting the extent and nature of tissue damage. Thus, we conducted a prospective analysis of the proteome of plasma EVs in patients referred to our center for suspected cardiac amyloidosis. We compared patients with a definitive diagnosis of ATTRwt and ATTRv (ATTR+ group) with patients suffering from nonamyloid heart failure (ATTR− group).

## Methods

### Ethics

The present analysis was based on blood samples collected as part of a research project entitled “Study of the myocardial microenvironment and toxicity of amyloid proteins in patients with cardiac amyloidosis”, which was approved by an institutional review board (CPP Sud-Méditerranée II, Marseille, France; approval references: 2021T2-12/2021-A00950-41 and 2022-A02416-37).

### Patients and blood sample collection

#### Study population and enrollment

Patients referred to the French National Reference Center for Cardiac Amyloidosis (Henri Mondor Hospital, Créteil, France) for suspected ATTR-CM were enrolled prospectively (ie, before initiation of disease-specific therapy). Inclusion was based on the presence of heart failure (with preserved or reduced ejection fraction) combined with at least 1 of the following: imaging features suggestive of cardiac amyloidosis (echocardiography or magnetic resonance imaging), positive diphosphonate scintigraphy, or a left ventricular wall thickness ≥12 mm (Table 1).

**Table 1:** Baseline Characteristics of the Study Population

	Whole Population (N = 65)	ATTR+ (n = 41)	ATTR− (n = 24)	*P* Value
Age, y, median [IQR]	77 [69-84]	82 [77-86]	70 [54-79]	0.12
Men, n (%)	48 (75)	30 (73)	18 (75)	0.60
Hereditary, n (%)		11 (26.8)		
Clinical features				
NYHA functional class III-IV, n (%)	21 (33)	13 (32.4)	8 (34)	0.34
HR (beats/min), median [IQR]	74 [68-89]	71 [67-89]	77 [68-91]	0.88
SBP (mm Hg), median [IQR]	127.0 [113-146]	129 [115-149]	117 [100-143]	0.56
Neurological involvement, n (%)	14 (21.8)	10 (24)	4 (16)	0.04
Biomarkers, median [IQR]				
Prealbumin (TTR, mg/L)	215 [173-267]	206 [172-251]	233 [193-279]	0.03
NT-proBNP (ng/L)	2,362 [1,377-4,757]	2,308 [1,331-4,900]	2,394 [1,430-4,873]	0.67
Troponin (ng/L)	62 [36-87]	72 [44-84]	54 [31-88]	0.12
eGFR (mL/min/m^2^)	55.4 [48.6-63.2]	56.8 [49.5-63.1]	53.8 [48.4-60.3]	0.09
AST (U/L)	23 [18-31]	21 [17-25]	30 [20-56]	0.07
ALT (U/L)	30 [24-34]	30 [25-33]	30 [23-46]	0.89
GGT (U/L)	63 [37-124]	56 [34-84]	81 [40-180]	0.04
CRP (mg/L)	3.7 [1.6-7.9]	4.2 [1.3-9.6]	3.7 [2.9-12.3]	0.04
Platelet count (g/L)	206 [170-244]	208 [176-246]	189 [159-245]	0.68
Echocardiographic features, median [IQR]				
LVEF (%)	47 [30-55]	50 [42-56]	38 [28-54.7]	0.02
LVEF <35%, n (%)	14 (22.5)	5 (12)	9 (37)	0.04
IVS thickness (mm)	15 [12-18]	17 [15-18]	12 [7-14]	0.03
GLS (% absolute value)	8.5 [6.2-10.7]	8.4 [6-17.5]	9.5 [8.0-13.0]	0.01
PASP (mmHg)	34 [28-42]	30 [28-45]	36 [30-47]	0.06
Medications, n (%)				
Anticoagulant	39 (61.8)	25 (62.5)	24 (58)	0.55
Furosemide	46 (72.5)	30 (73)	16 (66)	0.06
ACEI/ARB/MRA	44 (70)	20 (48)	24 (100)	0.01
ISGLT2	20 (33)	10 (22)	10 (41)	0.03
Betablocker	20 (31)	0	20 (83)	-
Amiodarone	13 (20.6)	8 (28.6)	5 (20)	0.06

Using Fisher exact test for categorical variables and the Mann-Whitney test for continuous variables, the threshold for statistical significance was set to *P* < 0.05.

ACEI = angiotensin-converting enzyme inhibitor; ALT = alanine aminotransferase; ARB = angiotensin II receptor blocker; AST = aspartate aminotransferase; ATTR = transthyretin amyloidosis; CRP = C-reactive protein; eGFR = estimated glomerular filtration rate; GGT = gamma-glutamyl transferase; GLS = global longitudinal strain; HR = heart rate; ISGLT = sodium-glucose transport 2 inhibitor; IVS = interventricular septum; LVEF = left ventricular ejection fraction; MRA = mineralocorticoid receptor antagonist; PASP = pulmonary artery systolic pressure; SBP = systolic blood pressure; TTR = transthyretin.

#### Diagnostic classification

Patients were assigned to 1 of 2 groups based on their final diagnosis. The ATTR+ group comprised patients with confirmed ATTR-CM, defined as ATTRwt or ATTRv, depending on TTR gene status. Diagnosis was established by positive diphosphonate scintigraphy (grade ≥2) or, in cases of grade 1 uptake or monoclonal gammopathy, by cardiac biopsy confirming amyloid deposition, in line with current expert consensus.^19^ The ATTR− group (control group) comprised patients with a final diagnosis of nonamyloid, noninfiltrative CM—specifically heart failure with preserved or reduced ejection fraction or hypertrophic CM. To minimize potential confounding, ATTR+ patients were frequency-matched to ATTR− patients approximately in a 2:1 ratio, based primarily on age, sex, and left ventricular ejection fraction. Group assignment was completed before EV analysis and no patient was excluded thereafter.

#### Exclusion criteria

Patients were excluded if they had: age under 18 years; a diagnosis of non-ATTR or another infiltrative CM (n = 89); treatment for malignancy or with immunosuppressants (n = 170); liver cirrhosis or renal dialysis (n = 31); and no data on the TTR genotype (n = 29) or no matching for age and sex (n = 46). All participants were diagnosed and followed up at our center. The detailed study flow chart is presented in Supplemental Data 1.

#### Clinical data collection

The following data were recorded at inclusion: personal medical history, ongoing medications, demographic and clinical variables (weight, height, heart rate, blood pressure, electrocardiogram, NYHA functional class, and cardiac and extracardiac events), and laboratory variables (high-sensitivity troponin, N-terminal pro-brain natriuretic peptide (NT-proBNP), creatinine, hepatic and renal biomarkers, proteinuria, C-reactive protein [CRP], plasma TTR, complete blood count, coagulation profile, and monoclonal gammopathy screening). Echocardiographic assessment included cardiac output and left ventricular parameters (Table 1).

#### Blood sample collection

At the first visit, 2 blood samples were collected in EDTA tubes. One milliliter of plasma was separated by centrifugation and immediately stored at −80 °C for subsequent EV isolation and proteomic analysis.

### EV isolation and characterization and the proteomics analysis

To generate a plasma rich in EVs, we used a previously described protocol^20^ that had been modified in accordance with guidelines from the International Society for Extracellular Vesicles.^21^, ^22^, ^23^ Briefly, EVs were isolated from plasma by ultracentrifugation and then characterized using flow cytometry and mass spectrometry-based proteomics (detailed protocol in Supplemental Data 2). To evaluate the enrichment of EV proteins, we compared our data with publicly available EV proteome libraries (ExoCarta July_2015_Release^24^, ^25^, ^26^ and/or Vesiclepedia August_2018_Release^27^^,^^28^), and measured the ratio between typical proteins of plasma (albumin) and EVs (CD9, flotillin-2). Downstream analyses were performed with gene ontology (GO), pathway and disease enrichment, all generated by StringApp (version 2.1.1)^29^ implemented in Cytoscape (version 3.9.1).^30^

### Statistical analysis

The groups’ clinical characteristics were compared using a *t*-test, chi-square test, or Mann-Whitney U test, depending on the data distribution.

A quantitative statistical analysis of proteomic data (obtained from the mean of 3 technical replicates for each patient) was based on an analysis of variance (Progenesis for the proteomics QI application, Waters) after the signal had been normalized against the internal standard (ENO-1 from yeast). Proteins with a *P* value ≤0.05, q-value (adjusted *P* value) ≤0.05, and an absolute log2 fold-change ≥1.25 were considered to be differentially expressed proteins (DEPs) and were included in subsequent analyses. Volcano plots, principal component (PC) analyses, receiver-operating characteristic (ROC) curves and bootstrap internal validation were performed using R software 4.5.0 within the RStudio environment. The clinical model for ROC curves consisted of age, sex, and estimated glomerular filtration rate, selected a priori as principal confounders available for all participants. No data-driven variable selection was used. Logistic regression model coefficients, ORs, and 95% CIs for each covariate are provided. Bootstrap internal validation (2,000 resamples) with optimism correction was used to provide bias-corrected area under the curve estimates for all models. Dot plots were built with GraphPad Prism 10.6. Heat maps were constructed using FunRich 3.1.3.^31^^,^^32^ To highlight relevant biological processes, pathways and diseases, enrichment analyses were performed using a false discovery rate threshold ≤0.05 in the Benjamini-Hochberg test.

## Results

### Clinical data

Between July 2020 and June 2024, 430 patients with suspected cardiac amyloidosis or a suspected, unrelated myocardiopathy consulted in our department (Supplemental Data 1). One hundred eleven patients met our criteria for inclusion in the ATTR + or ATTR− groups, and 65 patients with confirmed ATTR amyloidosis (ATTR+, n = 41) or without evidence of ATTR deposition (ATTR−, n = 24) were selected using frequency matching based on key demographic and clinical variables, including age, sex, and cardiac phenotype. Exact 2:1 matching was not enforced, to maximize the number of available cases while preserving group comparability for relevant covariates. The ATTR+ group comprised 30 ATTRwt cases and 11 ATTRv cases.

Demographic, clinical, laboratory, echocardiographic, and medication-related variables in the final cohort were compiled and analyzed statistically (Table 1). ATTR+ or ATTR− groups were well matched for most features. The similarity in NT-proBNP and troponin levels is not unexpected, as ATTR− patients were referred for suspected cardiac amyloidosis on the basis of unexplained cardiac hypertrophy and other clinical features suggestive of infiltrative CM. However, we observed differences in the plasma levels of gamma-glutamyl transferase and CRP, left ventricle and septum echocardiographic features, and medications (beta-blockers and drugs targeting the renin-angiotensin-aldosterone system).

### Characterization of plasma EVs

After enrichment from plasma, EVs were characterized using flow cytometry and liquid chromatography mass spectrometry/mass spectrometry (Supplemental Data 3). Flow cytometry enabled detection of EVs ranging from 160 to 900 nm in diameter (ie mostly resulting from plasma membrane budding), with smaller particles being below the laser resolution. The concentration and size distribution profile of ATTR+ and ATTR− EVs were similar. A large-scale proteomic analysis of all EVs enabled the quantification of 448 proteins. Most of these proteins were related to EVs, as indicated by the comparison with the Vesiclepedia^24^, ^25^, ^26^ and ExoCarta^27^^,^^28^ libraries. A GO analysis also showed a greater enrichment in proteins derived from the plasma membrane. The relative abundance of the plasma protein albumin compared with two EV markers (CD9 and flotillin-2) was measured and confirmed a major enrichment of EVs in the samples (6 orders of magnitude for both markers).

We finally characterized the putative source of the plasma EVs by mapping the tissue and blood distribution of the total protein content (Supplemental Data 4). Most of the quantified proteins were present in the blood, the heart, and the peripheral nervous system, which is commonly infiltrated by TTR amyloid fibrils.^31^

### The plasma EV protein signature in patients with ATTR-CM

Of the 448 quantified proteins in the ATTR+ vs ATTR− groups, 117 (26%) were DEPs: 85 were upregulated and 32 were downregulated in ATTR+ patients (Figure 1A, data set 1). A PC analysis based on individual patient levels of the 117 DEPs revealed a substantial separation between ATTR+ and ATTR− patients (with the first 2 dimensions of PCs accounting for 21.9% PC1 and 12.4% PC2 of the total variance, respectively) (Figure 1B). This finding was consistent with distinct proteomic profiles associated with disease status; which were also visible on a heatmap (Figure 1C). The dot plots (Figure 1D) highlight 6 significant DEPs in ATTR + EVs: TTR, plasmin(ogen) (the analysis cannot differentiate between plasmin and plasminogen), α2-antiplasmin, C1Q C-chain, complement factor B and complement factor D (CFD). Plasmin(ogen) and α2-antiplasmin potentially regulate amyloid fibril deposition directly (see subsequent section), whereas C1Q C-chain, complement factor B and CFD belong to the 6 complement proteins upregulated in ATTR+ EVs. Most importantly, although the ATTR+ vs ATTR− patients did not differ significantly with regard to the plasma TTR concentration (Table 1), TTR was very significantly upregulated in ATTR+ EVs (ratio ATTR+/ATTR− = 2.02; *P* < 0.00001; 95% CI: 1.69-2.64). Moreover, plasmin(ogen) was upregulated in ATTR+ EVs (ratio ATTR+/ATTR− = 1.58; *P* < 0.00001; 95% CI: 1.38-2.04) and α2-antiplasmin was downregulated in ATTR+ EVs (ratio ATTR+/ATTR− = 0.48; *P* < 0.0005; 95% CI: 0.34-0.75).

**Figure 1:**
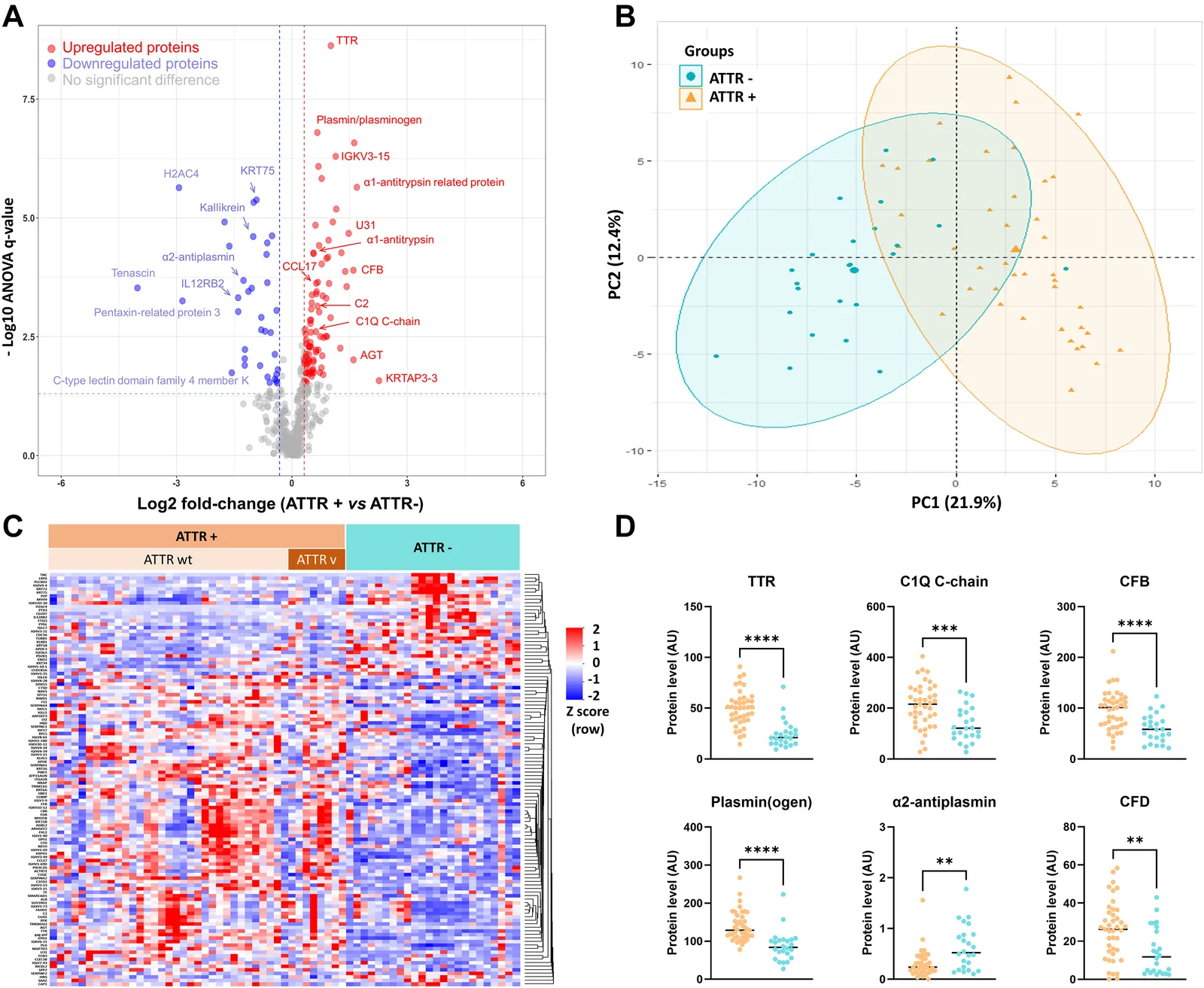
Quantitative Proteomics Data From Plasma Extracellular Vesicles in the ATTR+ and ATTR− Groups (A) A volcano plot depicting the log2-transformed fold-change and the corresponding -log10 adjusted *P* value (q-value) for all quantified proteins. (B) A principal component (PC) analysis score plot for the differentially expressed proteins. Each dot represents an individual’s proteomic profile projected onto the first two PC which represents the largest and second-largest sources of variance in the dataset. (C) Heat map of the 117 differentially expressed proteins. The color scale represents the range of log2-transformed values (blue, low expression; red, high expression). The dendrogram illustrates the clustering of protein expression based on similarity in expression patterns. (D) Individual raw patient data for levels of TTR, complement C1Q C-chain, CFB and CFD, plasmin(ogen), and α2-antiplasmin (alternate name: SERPINF2). ∗∗∗∗*P* < 0.0001, ∗∗∗*P* < 0.001, ∗∗*P* < 0.01 (multiple comparison with Welsh test). Data were obtained from 41 patients with confirmed ATTR amyloidosis (ATTR+) and 24 patients with no evidence of ATTR deposition (ATTR−). AGT = angiotensinogen; ANOVA = analysis of variance; ATTR = transthyretin amyloidosis; ATTRv = variant ATTR; ATTRwt = wild-type ATTR; AU = arbitrary units; CCL = C-C motif chemokine; CFB = complement factor B; CFD = complement factor D; IL12Rβ2 = IL12 receptor β2 chain; TTR = transthyretin.

ROC curves with bootstrap internal validation confirmed the discriminatory ability of TTR, plasmin(ogen) and α2-antiplasmin quantification in the EVs for distinguishing between ATTR+ and ATTR− patients (Figures 2A and 2B). TTR and plasmin(ogen) also added significant value to the clinical model (Figure 2C). Moreover, adjustment for baseline cardiovascular therapies supported the robustness of these findings (Supplemental Data 5). These analyses remain exploratory, and the diagnostic performance of the proposed biomarkers requires independent validation.

**Figure 2:**
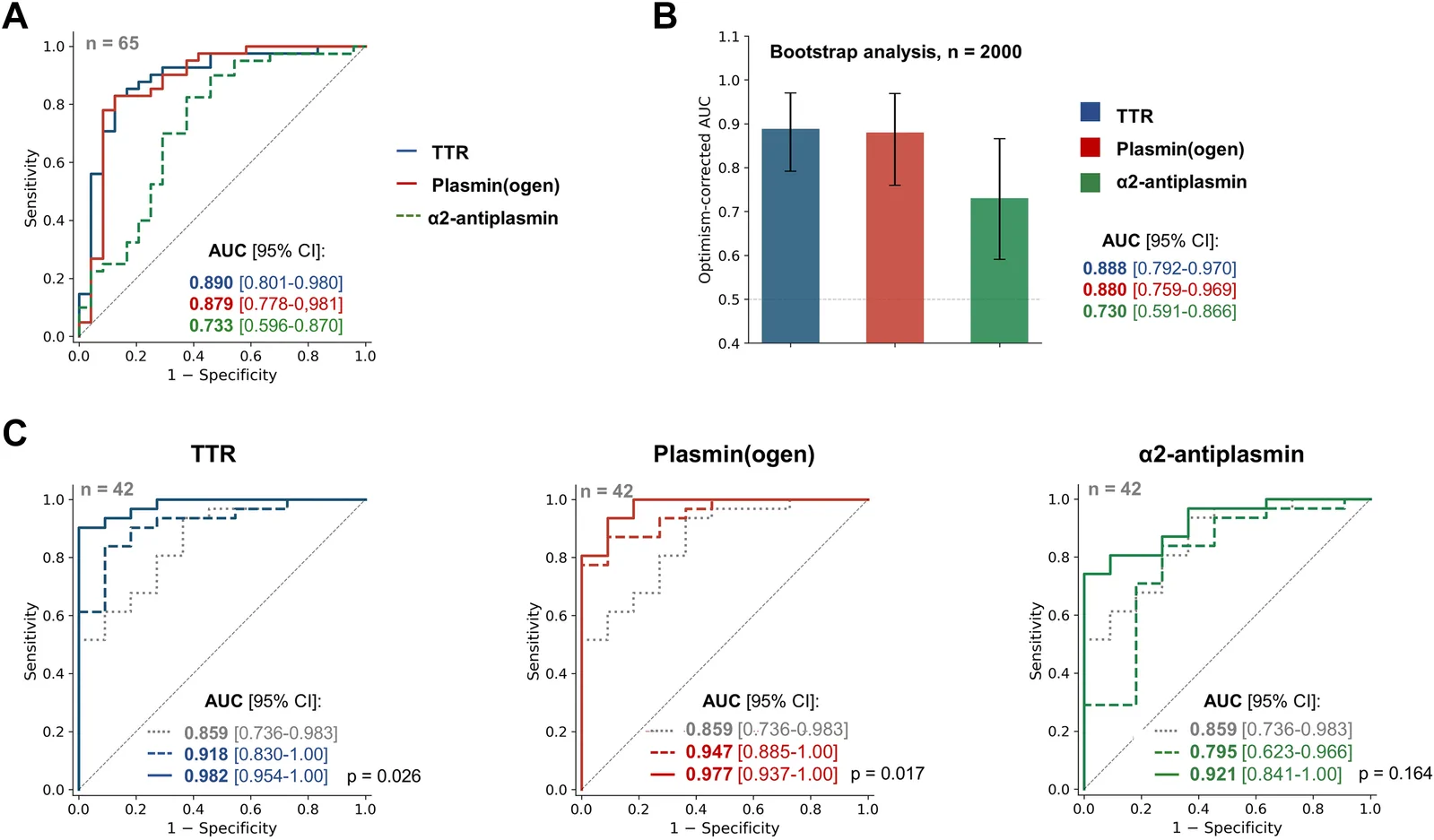
Receiver-Operating Characteristic Curves With Bootstrap Internal Validation and Clinical Adjustment Receiver-operating characteristic (ROC) curves were constructed to evaluate the ability of plasma extracellular vesicle (EV) levels of TTR, plasmin(ogen), and α2-antiplasmin to discriminate between ATTR+ and ATTR− patients. (A) Unadjusted ROC curves for the full cohort (n = 65 for TTR and plasminogen, n = 64 for α2-antiplasmin). (B) Bootstrap internal validation using 2,000 resamples performed on the full cohort. (C) ROC curves after adjustment for clinical covariates. *P* values compare the clinical model alone with the corresponding clinical model including each EV protein. Gray dotted line, clinical model alone; colored dotted line, EV protein alone (blue, TTR; red, plasmin(ogen); green, α2-antiplasmin); solid colored line, clinical model plus EV protein. AUC = area under the curve; other abbreviation as in Figure 1.

### GO, pathway and disease enrichment analyses

The GO and pathway enrichment analyses of 3 independent databases (GO Biological Processes, Reactome Pathways, and WikiPathways) confirmed the major importance of complement activation in ATTR+ EVs (Figure 3, Supplemental Data 2). Several other biological processes (mainly related to ECM remodeling, wound healing, coagulation, inflammation, and the humoral immune response) were significantly enriched among the DEPs. The disease enrichment analysis showed that proteins upregulated in ATTR+ EVs could be related to several affections; the most relevant for cardiac amyloidosis being “familial visceral amyloidosis” (Disease Ontology Identifier [DOID]: 005063), “coronary artery disease” (DOID: 3393) and “thrombosis” (DOID: 0060903). Familial visceral amyloidosis is a rare, inherited form of amyloidosis caused by mutations affecting various genes, including *TTR*. Overall, the pathways identified through these unsupervised, hypothesis-free analyses highlighted the importance of complement activation, deregulation of the coagulation cascade, and ECM remodeling.

**Figure 3:**
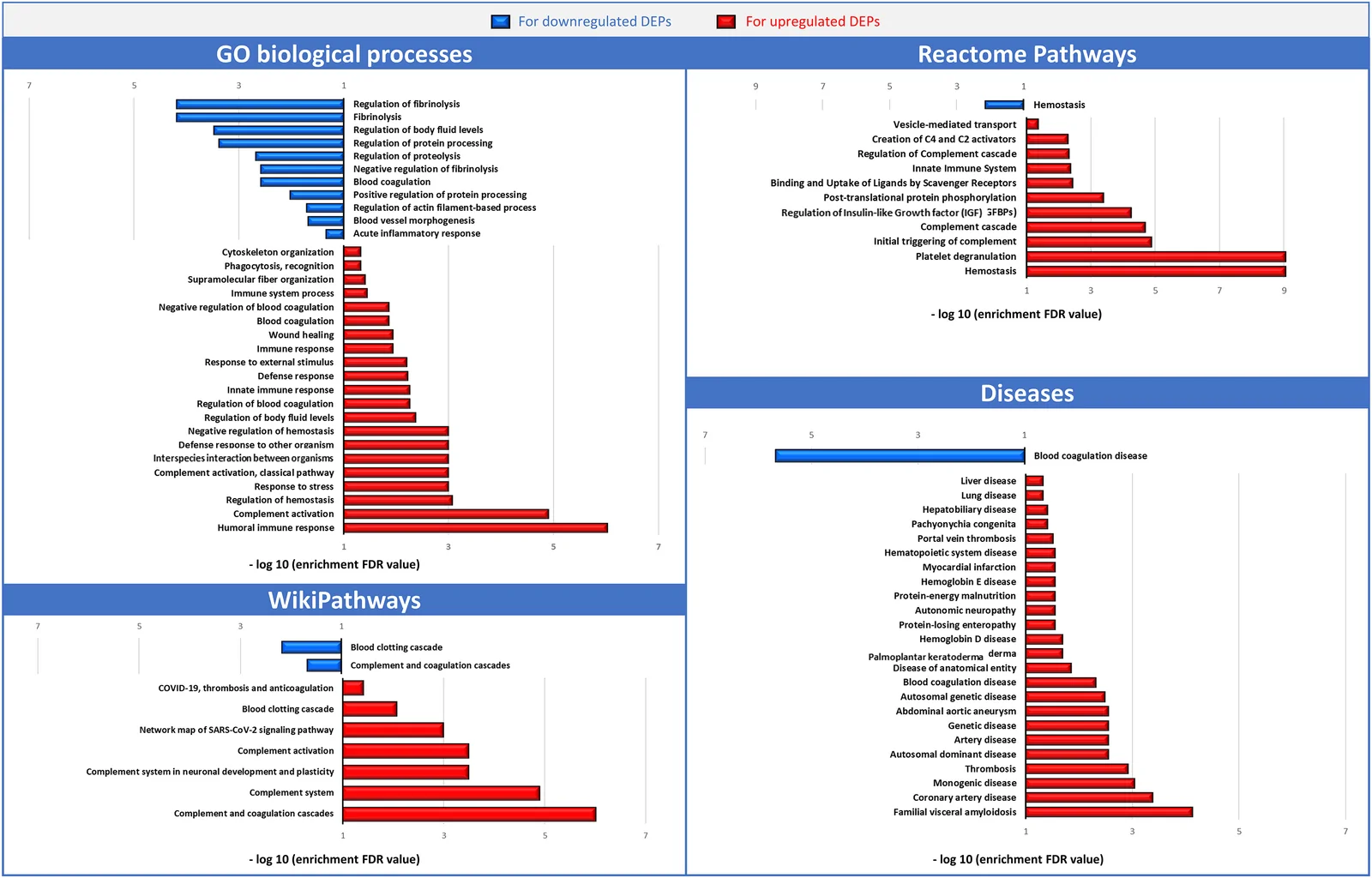
Enrichment Analyses of Differentially Expressed Proteins, Using Publicly Available Databases for Biological Processes and Diseases Analyses were performed using StringApp (version 2.1.1) implemented in Cytoscape (version 3.9.1). False discovery rate values were calculated as *P* values corrected from multiple testing within each category, using the Benjamini-Hochberg procedure. FDR = false discovery rate; GO = gene ontology.

### Plasmin/plasminogen is at the center of the ATTR+ protein signature

A closer examination revealed that several DEPs belonged to 4 molecular cascades: the complement system, the intrinsic (or contact) coagulation pathway, and the kallikrein-kinin, and renin-angiotensin systems (Supplemental Data 6, Figure 4A). Proteins from these cascades are functionally interconnected through activation and inhibition mechanisms, which frequently involve protease activity that generates bioactive peptides or enzymes from precursor proteins (Figure 4B).

**Figure 4:**
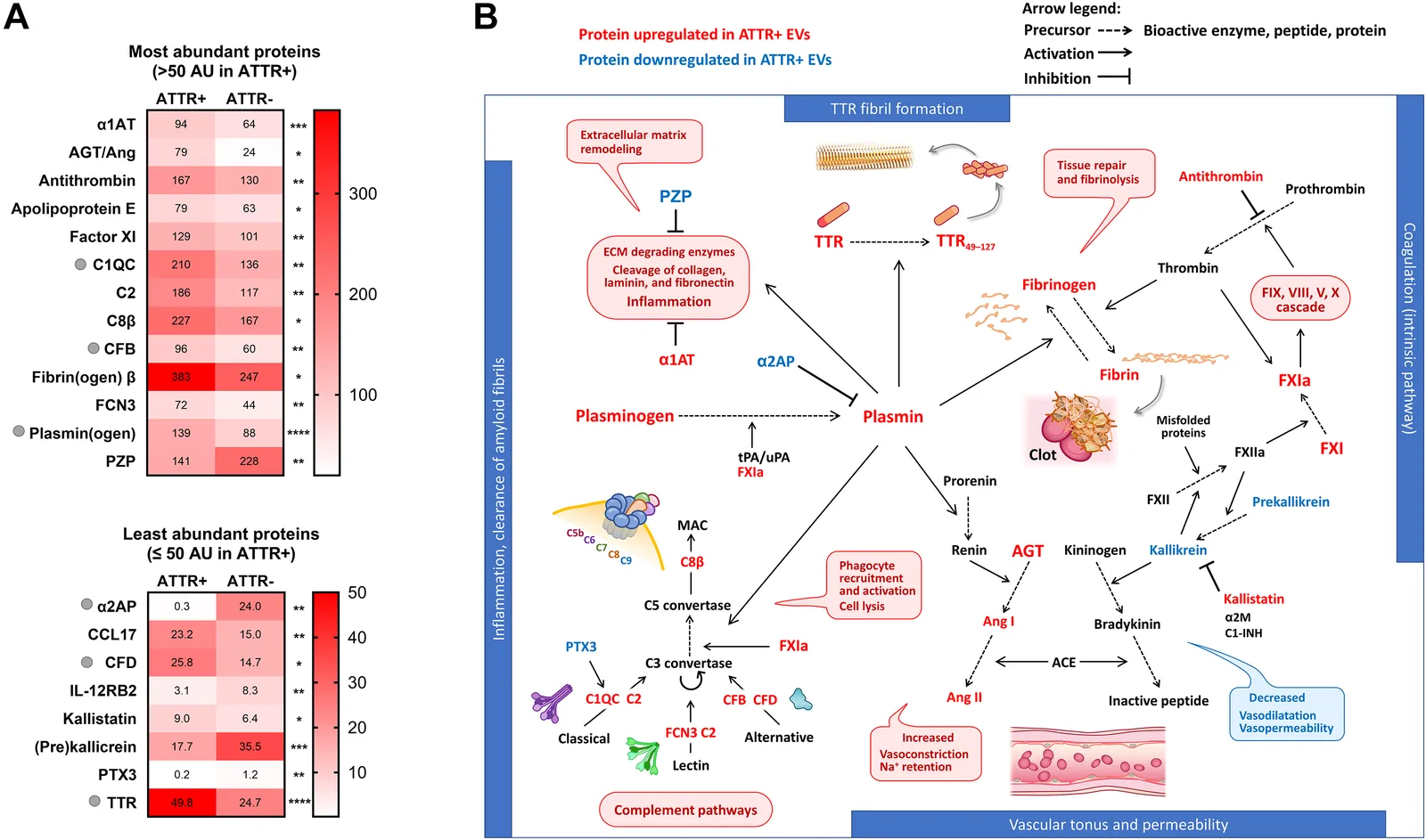
The Protein Network Underlying the ATTR+ Plasma Extracellular Vesicles’ Proteomic Signature (A) Plasma EV protein levels of TTR and Differentially Expressed Proteins from the complement system, the renin-angiotensin system, the kallikrein-kinin system, and the coagulation cascade, along with apolipoprotein E, IL-12 receptor β2 chain (IL12Rβ2) and C-C motif chemokine 17 (CCL17). Heat maps were constructed with quantitative values expressed as arbitrary units (AU). Each cell is labeled with the mean value; ∗q < 0.05; ∗∗q < 0.01; ∗∗∗q < 0.001; ∗∗∗∗q < 0.0001. Gray dots indicate differentially expressed proteins shown in Figure 1D. (B) The diagram highlights the main relationships between the various proteins involved in coagulation and tissue repair (the intrinsic pathway), inflammation (the complement system), and vascular homeostasis (the renin-angiotensin and kallikrein-kinin systems). α1AT = alpha-1 antitrypsin; α2AP = alpha-2 antiplasmin; α2M = α2 macroglobulin; ACE = angiotensin-converting enzyme; Ang I = angiotensin I; Ang II = angiotensin II; C1QC = complement C1q C chain; C1-INH = C1 inhibitor; C2 = complement C2; C8β = complement C8 β chain; EV = extracellular vesicle; FCN3 = ficolin 3; FXI (a) = coagulation factor XI (activated); FXII (a) = coagulation factor XII (activated); MAC = membrane attack complex; PTX3 = pentraxin 3; PZP = pregnancy zone protein; tPA/uPA = tissue plasminogen activator/urokinase-type plasminogen activator; other abbreviations as in Figure 1.

At the center of these pathways is the precursor plasminogen and its cleaved product plasmin; like other protein/peptide pairs, a proteomic analysis cannot distinguish between the 2. The levels of plasmin(ogen) were significantly elevated in ATTR+ EVs (ATTR+/ATTR− ratio = 1.58; *P* < 0.00001). The many key roles of this plasma protein (including interactions with protein targets and cell surface receptors, and the serine protease activity of the cleaved form plasmin) are well documented in the literature.^33^ In the context of the ATTR+ signature, plasmin(ogen) may interact with complement components, inflammatory cells, ECM-degrading enzymes, the renin-angiotensin system, and the coagulation cascade through its well-characterized fibrinolytic activity. Interestingly, plasmin can cleave TTR in vitro and in vivo to liberate the unstable TTR_49-127_ fragment and initiate the formation of amyloid fibrils.^34^^,^^35^ As we found that α2-antiplasmin (the main inhibitor of plasmin activity) was deeply downregulated in ATTR+ EVs (ratio ATTR+/ATTR− = 0.48; *P* = 0.00198), plasmin activity is probably enhanced. Overall, modifications of the plasma EV proteome in ATTR+ patients are in accordance with the known mechanisms of fibril formation and provide new insights into the pathophysiology of the disease.

## Discussion

The present study is the first to have explored the proteome of samples enriched in circulating EVs from patients with ATTR-CM. We identified a specific ATTR+ signature by comparison with patients with nonamyloid hypertrophic cardiac diseases (ie, the actual differential diagnosis for ATTR+ in clinical practice). Note that the screened population is not representative of a general cardiology population but rather of a highly selected tertiary-care referral cohort enriched with significant comorbidities. The ATTR+ signature comprised 117 DEPs, including TTR and 18 proteins directly involved in 4 highly intricate molecular systems: the complement system, the renin-angiotensin system, the kallikrein-kinin system, and the coagulation cascade (Figure 4). Some of the DEPs have been detected previously in the proteomic signatures of ATTR heart biopsies (apolipoprotein E and TTR) and carpal tunnel tissue (complement proteins).^7^^,^^36^, ^37^, ^38^ Based on unsupervised enrichment analyses, similar biological pathways were found to be prominent in EVs and cardiac tissue from ATTR patients. Some key proteins are discussed subsequently.

### TTR

This protein was significantly enriched in ATTR+ EVs, whereas corresponding plasma concentration was low. It has been proposed that TTR may be rapidly sequestered by amyloid aggregates in target organs, thereby preventing an increase in plasma concentrations.^10^ This may not be the case in EVs, the proteome of which tends to better reflect tissue-level processes.^39^^,^^40^ Gathering these previous works and our study, we can hypothetize that EVs derived from monocytes or macrophages might be enriched in TTR following the phagocytosis of amyloid deposits—a process potentially facilitated by complement activation.

### Complement system

Proteins from the complement cascade were among the most abundant in plasma EVs, as reported previously.^41^ Six complement components were specifically enriched in ATTR+ EVs, including CFD. CFD is mainly produced by adipocytes and mononuclear phagocytes (but not by the liver), circulates in an active form, and is highly substrate-specific.^42^ Importantly, CFD is a rate-limiting enzyme with a key role in amplifying C3 convertase activity. Thus, our results suggest that the complement cascade was fully activated and might have been amplified in the final steps by plasmin (Figure 4).^43^^,^^44^ Our results are in line with previous reports of irreversible ultrastructural damage and complement activation in cardiac tissue, carpal tunnel tissue, and subcutaneous abdominal fat tissue from ATTR patients.^36^^,^^37^^,^^45^

Conversely, complement activation might facilitate: 1) the elimination of cell debris and immune complexes;^46^ and 2) the clearance of amyloid deposits through immunoglobulin-mediated or direct binding of C1Q to amyloid fibrils.^47^

### Plasmin(ogen) and α2-antiplasmin

Plasminogen and/or plasmin were enhanced in ATTR+ EVs. We suggest that plasmin activity is elevated in ATTR+ EVs (due to a lower level of α2-antiplasmin) and that plasmin/plasminogen has a central role in connecting the pathways involved in the pathogenesis of amyloidosis. Although plasmin is well known for its fibrinolytic role, this serine protease has a broad substrate specificity and can therefore participate in ECM remodeling, activation of the renin-angiotensin system, and (as mentioned previously) complement activation.^46^^,^^48^ Moreover, plasminogen can bind to dead cells and misfolded proteins (including amyloid fibrils), and plasminogen receptors have notably been identified at the surface of immune cells and endothelial cells.^33^

Most importantly, plasmin can cleave TTR in vitro between residues 48 and 49 to liberate a truncated monomer (TTR_49-127_) that destabilizes the protein and promotes aggregation.^34^ This was confirmed for a mutant human TTR, using a transgenic mouse model of cardiac amyloidosis.^35^ Furthermore, deletion of the *Serpinf2* gene (encoding α2-antiplasmin) in this model accelerated the development of ATTR-CM.^35^ Thus, in ATTR+ EVs, upregulation of plasmin(ogen) and TTR, concomitant with a downregulation of α2-antiplasmin may reflect both active amyloidogenesis and an inflammatory response that eliminates dead cells, favor amyloid deposits, and facilitates tissue repair.

### The coagulation, kallikrein-kinin, and renin-angiotensin systems

Amyloid protofibrils have been shown to activate coagulation factor XII, initiating contact system signaling and contributing to inflammation and thrombosis—particularly in Alzheimer disease.^49^ Although this contribution has not yet been confirmed in ATTR, a similar mechanism might occur for TTR fibrils, potentially promoting vascular inflammation and thrombosis within the amyloid-laden myocardium. Indeed, coagulation factor XI and fibrinogen were strongly enhanced in ATTR+ EVs. Microvascular damage and thrombosis might be further accentuated by enhanced vasoconstriction, due to the imbalance between the renin-angiotensin and kallikrein-kinin systems in ATTR+ EVs. Our results are in line with reports of the frequent thrombotic and hemorrhagic complications of amyloidosis.^50^, ^51^, ^52^, ^53^, ^54^

### IL12 receptor β2 chain and C-C motif chemokine 17

Downregulation of IL12 receptor β2 chain (a key mediator of T helper type (Th)1 differentiation and interferon-gamma signaling) and greater expression of C-C motif chemokine 17 (a chemokine promoting Th2 cell recruitment) indicate a potential shift in immune polarization within the ATTR+ myocardial microenvironment. This particular environment might limit tissue damage and facilitate wound healing, as seen in the canonical Th2 responses driven by helminth infections.^55^ However, Th2 responses are also known to stimulate fibrosis.

Overall, the protein content of EVs did not phenocopy findings in whole plasma, as exemplified by TTR. The differential abundance of proteins previously isolated from myocardial tissue in ATTR-CM suggests that EVs can be released by cells activated or stressed by amyloid deposition, such as cardiomyocytes, endothelial cells, and phagocytes. Thus, the EV content might represent a signature of tissue remodeling and cellular dysfunction indicative of the disease stage.

### Study limitations

Although no EV isolation method achieves complete purity, ultracentrifugation minimizes the co-enrichment of plasma contaminants.^56^^,^^57^ In our study, EV purity reached approximately 87% with 6 orders of magnitude enrichment for EV protein markers.

Although demographic, clinical, biomarker, and echocardiographic matching was carefully checked, differences between ATTR+ and ATTR− persisted for certain variables: biomarkers (gamma-glutamyl transferase and CRP), left ventricle echocardiographic features, and medications. The subtle difference of CRP concentrations (both in the reference range) might have reflected a slightly greater inflammation associated with amyloid deposition (consistent with the elevated levels of complement proteins observed in ATTR+ EVs). Adjusting for echocardiographic severity (left ventricular ejection fraction, interventricular septum, and global longitudinal strain) was avoided, as these variables lie within the causal pathway of ATTR and may themselves result from amyloid deposition, risking overadjustment. Regarding medications, beta-blockers are not routinely used in ATTR+ patients in the absence of a separate indication, which was reflected in our cohort. The more frequent use of angiotensin-converting enzyme inhibitors, angiotensin II receptor blockers, and mineralocorticoid receptor antagonists in ATTR− patients might have been a confounding factor in our analysis of plasmin(ogen), the proteins from the renin-angiotensin and kallikrein-kinin systems. Nevertheless, adjustment for cardiovascular therapies did not materially affect the observed associations, although residual confounding cannot be completely excluded. Therefore, the candidate protein biomarkers constituting the ATTR+ signature will require validation in larger independent cohorts of patients to confirm their discriminatory performance. In addition, future studies should determine the tissue and cellular origin of the EVs carrying these candidate biomarkers.

## Conclusions

This proteomic study of plasma EVs in ATTR-CM identified a disease-specific signature of DEPs (Central Illustration). Beyond confirming the enrichment of EV-associated TTR—despite low circulating plasma levels—our findings reveal a functionally interconnected protein network centered on plasmin/plasminogen, linking dysregulated fibrinolysis, complement activation, and vascular inflammation to the pathophysiology of ATTR. Collectively, these results suggest that plasma EVs capture molecular changes that are not detectable through conventional biomarkers.

Perspectives **COMPETENCY IN MEDICAL KNOWLEDGE**: Although circulating biomarkers such as TTR, NT-proBNP, and troponin can provide clinically useful information, their diagnostic utility remains limited compared with established diagnostic modalities. In particular, they cannot substitute for bone tracer scintigraphy, which remains central to the noninvasive diagnosis of ATTR. Moreover, complementary approaches are needed to gain further insight into the underlying molecular mechanisms of disease.

**TRANSLATIONAL OUTLOOK**: Beyond improving our understanding of disease biology, the analysis of EV-derived proteins may open new avenues for biomarker discovery through methods compatible with routine laboratory practice. In particular, EV-associated TTR and plasmin(ogen) warrant further investigation as potential adjuncts to current diagnostic algorithms. If independently validated, such biomarkers could help refine the noninvasive evaluation of patients with suspected cardiac amyloidosis. Achieving this goal will require standardized EV enrichment methods suitable for routine laboratory use, as well as a better understanding of the cellular origin and biological significance of these EV-associated proteins in ATTR-CM.

**Central Illustration:**
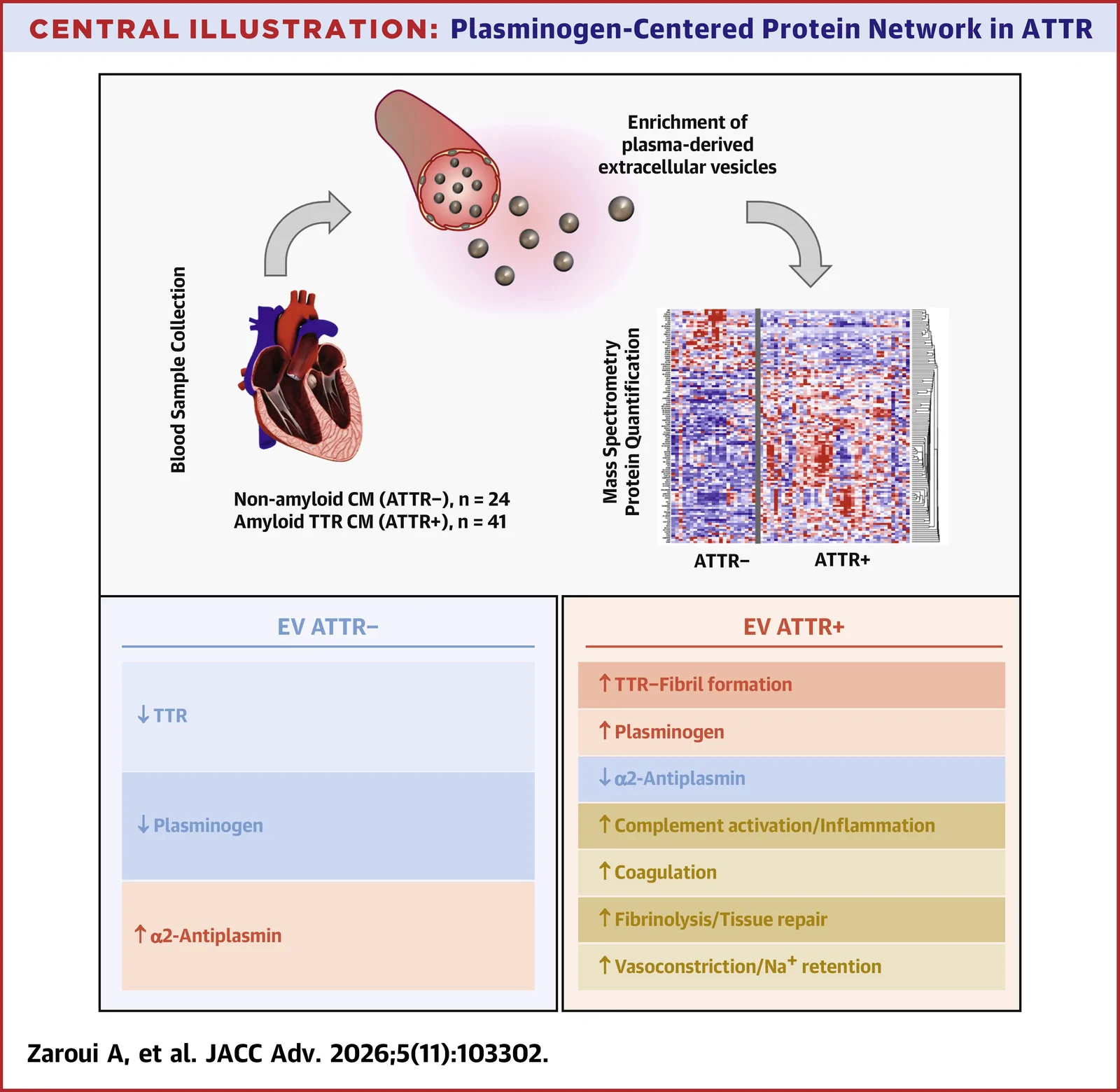
Plasminogen-Centered Protein Network in ATTR We prospectively isolated plasma EVs from ATTR cardiomyopathy patients (ATTR+) and nonamyloid cardiomyopathy controls (ATTR–), and then profiled them by quantitative liquid chromatography mass spectrometry/mass spectrometry proteomics. ATTR + EVs showed a distinct signature enriched in vascular homeostasis, coagulation, and inflammation proteins, centered on an interconnected plasmin/plasminogen network. Notably, transthyretin and plasminogen were elevated while alpha2-antiplasmin, its inhibitor, was reduced—an imbalance of particular relevance given plasmin's known role in promoting amyloidogenesis through transthyretin cleavage. CM = cardiomyopathy; EV = extracellular vesicle; TTR = transthyretin.

## Funding support and author disclosures

This work was funded by Assistance Publique-Hôpitaux de Paris (Paris, France), University Paris-Est-Créteil (Créteil, France), Association pour la Recherche Multi-Disciplinaire en Cardiologie (Créteil, France) and Université Grenoble Alpes (Saint-Martin-d'Hères, France). The funders had no role in the study design, sample and data collection, analysis, interpretation of data, or the writing of the publication. Dr Zaroui has received consultant fees and/or research grant from Alexion, Alnylam, AstraZeneca, Bayer, Pfizer, NovoNordisc, and Neurimmune. Dr Damy has received consultant fees and/or, research grant from Alexion, Alnylam, Astra-Zeneca, Bayer, Pfizer, NovoNordisc, and Neurimmune. All other authors have reported that they have no relationships relevant to the contents of this paper to disclose.
