# Supplementary material for: Quantitative Proteomics of Plasma-Derived Extracellular Vesicles Reveals a Plasminogen-Centered Protein Network in Transthyretin Cardiac Amyloidosis
Source: JACC Adv. 2026 Oct 8;5(11):103302. doi: 10.1016/j.jacadv.2026.103302 (PMC13675698; doi:10.1016/j.jacadv.2026.103302)
Supplement: Supplementary file 1 [file mmc1.docx]

# Quantitative Proteomics of Plasma-Derived Extracellular Vesicles Reveals a Plasminogen-Centred Protein Network in Transthyretin Cardiac Amyloidosis

Zaroui A et al.

**Datasets and Supplemental data**

# dataSET 1. Quantitative proteomic data ATTR+ *versus* ATTR-

# dataSET 2. String enrichment for Up/Down-DEP in ATTR+ *versus* ATTR-

See Excel files attached

# Supplemental data 1. STUDY Flowchart

# Supplemental data 2. Detailed material and methods

## 1. Extracellular vesicle (EV) isolation

The protocol is based on the enrichment of EVs prior to characterization by FACS and proteomics, as described previously with some modifications^1^ and in line with the MISEV guidelines.^2,3^ Briefly, 1 mL of plasma was centrifuged at 200 g for 5 min and then at 2,000 g for 10 min, to remove cell debris. Supernatant fractions were centrifuged at 12,000 g for 30 min. EVs were obtained from the supernatant by centrifugation at 120,000g for 90 min (Beckman Ti50). The resulting pellets were resuspended in a large volume of ultrapure water filtered through a 220-nm filter, and then washed and collected by ultracentrifugation at 120,000 g for 90 min. All the steps were performed at 4°C. EV-containing pellets were resuspended in 200 μL of ultrapure water and kept overnight at 4°C. The concentration of EV proteins was determined using a micro-BCA Protein Assay Kit (Thermo Fisher Scientific, Waltham, MA, USA). The EV preparations were stored at -80°C to achieve more stability compared to 20°C.

## 2. Flow cytometry analysis of EVs

EVs with a diameter of at least 160 nm (corresponding to microparticles, which are derived from budding of the plasma membrane) were characterized by flow cytometry on an LSR Fortessa ﬂow cytometer with a small-particle option (BD Biosciences, San Jose, CA) based on photomultiplier (PMT)-coupled forward scatter (FSC). EV flow cytometry assays were performed in accordance with the guidelines of the International Society for Extracellular Vesicles.^4^ The sensitivity of this cytometer was tested with Megamix and Spherotech latex beads, as well as ApogeeFlow silica beads. ^5^ Calibration and the absence of background noise from the machine in the window were checked for each test.

The number of EVs was determined using Trucount™ beads, as previously described.^1^ EVs were identified by CD9 labeling. Megamix-Plus ﬂuorescent beads (Biocytex, La Ferté Saint-Aubin, France) were used to differentiate EVs according to their size (diameters of 160-200 nm, 200-240 nm, 240-300, 300-500 nm and 500-900 nm). The ﬂow cytometry data were analyzed with FlowJo software (version 10.7.1). All statistical analyses were performed with GraphPad Prism 10.6. ATTR+ and ATTR- EV numbers were compared by the Mann Whitney test.

## 3. Label-free quantitative proteomic analysis

The EV proteome was identified and quantified using a differential label-free mass-spectrometry approach based on a bottom-up protocol with a nano-liquid chromatography mass spectrometry (LC-MSE) analysis (Figure S1).^1,6^ In details, 30 μg of isolated EVs were diluted in 50 mM ammonium bicarbonate pH 7.8 with Rapigest (Waters, Milford, MA, USA). Next, reduction, alkylation and digestion were performed with dithiothreitol, iodoacetamide, and trypsin, respectively. Tryptic digestion was performed overnight at 37°C with a trypsin/protein ratio of 3%. Formic acid (FA) was added to each sample (final concentration: 0.3%) to stop the trypsin digestion and obtain pellets. The supernatant (containing desalted peptides) was retrieved and dried, using a Speed-Vac. Dried samples were diluted in buffer A (H_2_O with 0.1% FA) and analyzed using nano-LC-MS on a NanoAcquity-ESI-SynaptG2-Si system (Waters) operating in nano-ESI positive mode. After loading onto the UPLC peptide BEH130 C18 nanoACQUITY^TM^ column (100 μm x 100 mm, diameter: 1.7 μm diameter; Waters) at a flow rate of 0.45 μL/min, peptides were separated with a gradient increasing from 1% Buffer B (acetonitrile with 0.1% FA)/99% Buffer A (H_2_O with 0.1% FA) to 40% Buffer B/60% Buffer A, and then to 85% Buffer B/15% Buffer A, over 120 min. MS spectra were recorded in a 50-2,000 *m/z* mass range at a resolution of 10,000-20,000. The nano LC-MS method included 120 min of acquisition time, positive polarity, MS acquisition range of 50-4,000 Da, and a collision energy range of 20-55 V. Proteins were identified and quantified using Progenesis software for proteomics QI (Waters), with the ion accounting algorithm and the following parameters: trypsin digestion enzyme; a maximum of two missed cleavages; maximum protein mass: 250 kDa; fixed modification: carbamodomethyl (C); variable modifications: oxidation (M), deamination (N, Q) and acetylation (N-ter); false discovery rate: 1%, automatic mass tolerance, isotope filter: 2; *Homo sapiens* UniprotKB/Swissprot database (canonical proteins from UP000005640 proteome Release-2021-02; https://www.uniprot.org/) concatenated with the sequence of angiotensin II (DRVYIHPF) and the internal standard (ENO1_YEAST, P00924). The proteomic data were A analyzed quantitatively in an analysis of variance, using the Progenesis for proteomics QI application (Waters). We focused on the three most abundant and confidently identified unique peptides (non-conflicting peptides) by considering the correlation factor of each and normalizing the signal against the ENO-1 internal standard. Differential quantitative data were visualized in a volcano plot (**DATASET 1**). The fold-change threshold of 1.25 was selected based on the volcano plot distribution, where this value corresponded to the inflection point separating the bulk of proteins showing random, non-significant variation from a distinct subset exhibiting both consistent-magnitude and statistically significant changes. Therefore, only proteins with p- and q-value ≤0.05 and a FC>1.25 were considered to be significantly deregulated. These criterions allowed to capture proteins with smaller but reproducible and statistically robust changes for low abundance regulatory proteins or signaling proteins that may nonetheless carry biological significance in EV. These differentially expressed proteins were included in the EV-ATTR signature. Note that values of zero abundance, corresponding to features not detected or not aligned across LC-MS runs, were treated as missing. Overall, missing values represented 1% of the entire dataset, with a mean missing rate of 1% across both patients and proteins, and a 95th percentile of 8% across proteins. We confirmed that all proteins retained in the protein signature met this 8% threshold. Within this signature, the mean missing-value rate was 0.4%, with a 95th percentile of 2% and a maximum of 6%. These residual missing values were handled on a pairwise basis in subsequent statistical analyses, without imputation.

**Figure S1. Shotgun proteomics workflow.**

## 4. Downstream analysis of proteomic data

To check the proportion of proteins known to be found in EVs, the experimental data were compared with EV libraries (ExoCarta, July_2015_Release^7–9^ and/or Vesiclepedia, August_2018_Release.^10,11^ These results were plotted as **Venn diagrams**, using FunRich software (version 3.1.3).^12,13^ We also measured the ratio between typical proteins of plasma (albumin) and EVs (CD9, flotillin-2).

To reduce the dimensionality of the dataset for each quantified protein from the 65 samples to two dimensions and determine whether or not the protein profiles from ATTR+ and ATTR- patients forms two distinct clusters, we performed a **principal component analysis** on the EV-ATTR signature using R (version 4.2.2) with the ggfortify and ggplot2 packages and the “fviz_pca_ind” function.

The **heat map** was plotted using FunRich software (version 3.1.3) ^12,13^. Z-scores were obtained by subtracting the mean and dividing by the standard deviation.

To generate both **EV-ATTR network** and **GO/pathway enrichment** analyses, we used StringApp v2.1.1 ^14^ implemented in Cytoscape v3.9.1 ^15^. The STRING network of the EV-ATTR signature was clustered through the MCL cluster network, with a granularity parameter of 4. A gene ontology (GO) enrichment analysis was performed with *Homo sapiens* database as the background (Table S2). The GO cellular compartment enrichment analysis was performed for all the identified proteins, while the GO biological processes and pathways enrichment analyses were performed for the EV-ATTR signature STRING network only (i.e. significantly deregulated proteins in ATTR *vs.* non-amyloid cardiomyopathy). FDR values (False discovery Rates) for String Enrichment analysis were calculated as p-values corrected for multiple testing within each category using the Benjamini–Hochberg procedure.

# Supplemental data 3. EV charaCteriZation and purity

**Figure S3. Characterization of EVs derived from plasma from ATTR+ and ATTR- patients.** (**A**) The number of plasma microparticles (containing the largest EVs, diameter ≥ 160 nm) was assessed by flow cytometry, using Trucount beads. EVs were identified by CD9 labeling, in line with MISEV recommendations for tetraspanin-based EV characterization. Note that CD9^+^ EVs likely represent a subset of the total circulating EV population rather than the full EV pool. Also, EVs smaller than 160 nm could not be captured by the flow cytometry technique, while they may contain relevant biological information. However, our mass spectrometry-based proteomic approach is not constrained by particle size and enables the detection of EV-associated proteins across the full spectrum of vesicle subtypes (**B**) Megamix ﬂuorescent beads were used to differentiate particles with diameters of 160-200 nm, 200-240 nm, 240-300, 300-500 nm and 500-900 nm. Data were analyzed with the FlowJo software (version 10.7.1). (**C**) Venn diagram representing the quantified proteins found in the Vesiclepedia^10,11^ and/or ExoCarta^7^ EV libraries. Eighty seven percent (87%) of the quantified proteins that could be mapped had already been reported in Vesiclepedia^10,11^ and/or ExoCarta^7^ EV libraries. (**D**) The top 10 extracellular and cellular compartments in the GO enrichment analysis, using StringApp v2.1.1 implemented in Cytoscape v3.9.1. Most of the proteins were related to EVs. Ranked by significance, the top 10 extracellular/cellular compartments (all with a p-value below 0.00001) were as follows: extracellular space, blood microparticle, extracellular region, secretory granule lumen, vesicle, EV, extracellular membrane-bounded organelle, secretory granule, fibrinogen complex, and platelet alpha granule. (**E**) The ratio of albumin (most abundant protein in the plasma) relative to two recognized markers of EVs (Flotilin-2 [Flot-2] and CD9)^16^ was measured in ATTR+ and ATTR- patient samples after EV isolation and compared with the expected value in raw plasma. The latter was calculated using a value of 40 g/L for albumin concentration and values of 1.2x10^-6^ g/L and 1.7x10^-7^ g/L reported in The Human Protein Atlas ^17,18^ for Flot-2 and CD9 concentrations, respectively.

# Supplemental data 4. Distribution enrichment analysis of THE EV-ATTR Protein Signature IN Tissues and Body Fluids.

Circulating EVs represent a complex and heterogeneous system, as they can be derived from any cell or tissue in the body. We tried to characterize the source of the plasma EVs by mapping the tissue and blood distribution of total proteins and the differentially expressed proteins (DEPs) (Figure S4A and B, respectively). Most of the proteins were present in the blood, the heart, and several other organs commonly infiltrated by amyloid fibrils, such as the peripheral nervous system, liver, and kidney.^19^ Thus, plasma EV proteins might reflect the protein content of the main target organs in the amyloidogenic process.

**Figure S4.** Heat maps representing the landscape of tissue/blood cell origins among plasma EV samples for (A) all quantified proteins (337 of the 448 proteins mapped in STRING), (B) EV proteins in the ATTR signature (89 of the 117 of DEPs mapped in STRING). The heat maps were plotted using Funrich software and STRING data (StringApp v2.1.1). The Z-score was calculated from the STRING confidence score from the Compartments database (https://compartments.jensenlab.org/search); a higher score means a higher degree of confidence.).

# Supplemental data 5.

| EV protein | Subgroup | N | Odds Ratio (95% CI) | P value |
| --- | --- | --- | --- | --- |
| TTR | All patients with drug data | 37 | 2.03 × 10^53^ (0.00–∝) | 0.999 |
| TTR | Patients receiving HF therapies | 34 | 2.55 × 10^53^ (0.00–∝) | 0.999 |
| Plasminogen | All patients with drug data | 37 | 6.33 × 10^17^ (0.00–3.28 × 10^78^) | 0.566 |
| Plasminogen | Patients receiving HF therapies | 34 | 6.33 × 10^17^ (0.00–3.28 × 10^78^) | 0.566 |
| α2-antiplasmin | All patients with drug data | 37 | 0.11 (0.02–0.71) | 0.020 |
| α2-antiplasmin | Patients receiving HF therapies | 34 | 0.14 (0.02–0.80) | 0.027 |

**Supplementary Table. Sensitivity analyses according to background heart failure (HF) therapies.** Sensitivity analyses evaluating the association between EV-associated proteins and ATTR cardiomyopathy after restricting the ATTR+ cohort to patients with available medication data and to patients receiving heart failure therapies. Odds ratios and 95% confidence intervals (CIs) were derived from logistic regression models. The persistence of significant associations across these restricted analyses, with the exception of a slight effect of treatment on α2-antiplasmin, supports the robustness of the primary findings and suggests a limited impact of background cardiovascular therapies on the observed EV proteomic signatures.

**Figure S5. Impact of background HF therapies on the diagnostic performance of EV-associated proteins for ATTR cardiomyopathy.** Receiver operating characteristic (ROC) curves comparing unadjusted models (dashed line) and models additionally adjusted for background cardiovascular therapies (solid line), including angiotensin-converting enzyme inhibitors (ACEi), angiotensin receptor blockers (ARB), and mineralocorticoid receptor antagonists (MRA). Analyses were performed in the subset of patients with complete medication data (n = 37; ATTR+ n = 30, ATTR− n = 7). Diagnostic performance remained largely unchanged after adjustment, with similar areas under the curve (AUCs) for EV-associated TTR (blue; protein alone AUC = 0.915 [95% CI 0.826–1.000], TTR + treatment AUC 1.000 [95% CI 0.994–1.000]; p = 0.059), plasminogen (red; protein alone AUC = 0.945 [95% CI 0.882–1.000], plasminogen + treatment AUC 1.000 [95% CI 0.994–1.000]; p = 0.087), and α2-antiplasmin (green; protein alone AUC 0.793 [95% CI 0.619–0.968], α2-antiplasmin + treatment AUC 0.915 [95% CI 0.833–1.000]; p = 0.149), indicating that the observed EV proteomic signatures were not substantially influenced by differences in background heart failure therapies. All p-values compare protein alone vs protein + treatment (bootstrap permutation test). eGFR, estimated glomerular filtration rate; TPR, true positive rate; FPR, false positive rate; ns, non-significant.

# Supplemental data 6.

**Proteins up- or downregulated in ATTR EVs in the complement and coagulation cascades (WP558 - Homo sapiens - version 2024-07-22) and the blood clotting cascade (WP272 - Homo sapiens - version 2023-11-25)**

(wikipathway: https://www.wikipathways.org/pathways). Proteins upregulated in EVs from ATTR patients (relative to nonamyloid cardiomyopathy patients) are highlighted in red, while the proteins that are downregulated in ATTR patients are highlighted in blue.

REFERENCES

1.	Ferrara B, Bourgoin-Voillard S, Habert D, Vallée B, Nicolas-Boluda A, Simanic I, Seve M, Vingert B, Gazeau F, Castellano F, et al. Matrix stiffness regulates the protein profile of extracellular vesicles of pancreatic cancer cell lines. *Proteomics*. 2024;e2400058.

2.	Théry C, Witwer KW, Aikawa E, Alcaraz MJ, Anderson JD, Andriantsitohaina R, Antoniou A, Arab T, Archer F, Atkin-Smith GK, et al. Minimal information for studies of extracellular vesicles 2018 (MISEV2018): a position statement of the International Society for Extracellular Vesicles and update of the MISEV2014 guidelines. *J Extracell Vesicles*. 2018;7:1535750.

3.	Welsh JA, Goberdhan DCI, O’Driscoll L, Buzas EI, Blenkiron C, Bussolati B, Cai H, Di Vizio D, Driedonks TAP, Erdbrügger U, et al. Minimal information for studies of extracellular vesicles (MISEV2023): From basic to advanced approaches. *Journal of Extracellular Vesicles*. 2024;13:e12404.

4.	Welsh JA, Arkesteijn GJA, Bremer M, Cimorelli M, Dignat-George F, Giebel B, Görgens A, Hendrix A, Kuiper M, Lacroix R, et al. A compendium of single extracellular vesicle flow cytometry. *J Extracell Vesicles*. 2023;12:e12299.

5.	Delorme AS, Laguide A, Tamagne M, Pinheiro MK, Cagnet L, Neyrinck-Leglantier D, Khelfa M, Cleophax S, Pirenne F, Vingert B. Immune interactions and regulation with CD39+ extracellular vesicles from platelet concentrates. *Front. Immunol.* [Internet]. 2024 [cited 2026 Jan 29];15. Available from: https://www.frontiersin.org/journals/immunology/articles/10.3389/fimmu.2024.1397967/full

6.	Blangy-Letheule A, Vergnaud A, Dupas T, Habert D, Montnach J, Oulehri W, Hassoun D, Denis M, Lecomte J, Persello A, et al. Value of a secretomic approach for distinguishing patients with COVID-19 viral pneumonia among patients with respiratory distress admitted to intensive care unit. *J Med Virol*. 2024;96:e29756.

7.	Keerthikumar S, Chisanga D, Ariyaratne D, Al Saffar H, Anand S, Zhao K, Samuel M, Pathan M, Jois M, Chilamkurti N, et al. ExoCarta: A Web-Based Compendium of Exosomal Cargo. *Journal of Molecular Biology*. 2016;428:688–692.

8.	Mathivanan S, Fahner CJ, Reid GE, Simpson RJ. ExoCarta 2012: database of exosomal proteins, RNA and lipids. *Nucleic Acids Res*. 2012;40:D1241–D1244.

9.	Mathivanan S, Simpson RJ. ExoCarta: A compendium of exosomal proteins and RNA. *PROTEOMICS*. 2009;9:4997–5000.

10.	Pathan M, Fonseka P, Chitti SV, Kang T, Sanwlani R, Van Deun J, Hendrix A, Mathivanan S. Vesiclepedia 2019: a compendium of RNA, proteins, lipids and metabolites in extracellular vesicles. *Nucleic Acids Research*. 2019;47:D516–D519.

11.	Kalra H, Simpson RJ, Ji H, Aikawa E, Altevogt P, Askenase P, Bond VC, Borràs FE, Breakefield X, Budnik V, et al. Vesiclepedia: A Compendium for Extracellular Vesicles with Continuous Community Annotation. *PLoS Biol*. 2012;10:e1001450.

12.	Pathan M, Keerthikumar S, Ang C-S, Gangoda L, Quek CYJ, Williamson NA, Mouradov D, Sieber OM, Simpson RJ, Salim A, et al. FunRich: An open access standalone functional enrichment and interaction network analysis tool. *PROTEOMICS*. 2015;15:2597–2601.

13.	Fonseka P, Pathan M, Chitti SV, Kang T, Mathivanan S. FunRich enables enrichment analysis of OMICs datasets. *Journal of Molecular Biology*. 2021;433:166747.

14.	Doncheva NT, Morris JH, Gorodkin J, Jensen LJ. Cytoscape StringApp: Network Analysis and Visualization of Proteomics Data. *J Proteome Res*. 2019;18:623–632.

15.	Lotia S, Montojo J, Dong Y, Bader GD, Pico AR. Cytoscape App Store. *Bioinformatics*. 2013;29:1350–1351.

16.	Welsh JA, Goberdhan DCI, O’Driscoll L, Buzas EI, Blenkiron C, Bussolati B, Cai H, Di Vizio D, Driedonks TAP, Erdbrügger U, et al. Minimal information for studies of extracellular vesicles (MISEV2023): From basic to advanced approaches. *J Extracell Vesicles*. 2024;13:e12404.

17.	Uhlén M, Karlsson MJ, Hober A, Svensson A-S, Scheffel J, Kotol D, Zhong W, Tebani A, Strandberg L, Edfors F, et al. The human secretome. *Sci Signal*. 2019;12:eaaz0274.

18.	The human blood atlas - The Human Protein Atlas [Internet]. [cited 2026 Feb 17];Available from: https://www.proteinatlas.org/humanproteome/blood

19.	Feitosa VA, Neves PDMM, Jorge LB, Noronha IL, Onuchic LF. Renal amyloidosis: a new time for a complete diagnosis. *Braz J Med Biol Res*. 2022;55:e12284.
